# Prognostic risk model based on cholesterol metabolism–Associated gene module for idiopathic pulmonary fibrosis

**DOI:** 10.1371/journal.pone.0345310

**Published:** 2026-04-09

**Authors:** Jia Zhang, Linshu Xie, Xinyu Yang, Xiaoli Wang, Qianqian Liu, Xiaoju Zhang

**Affiliations:** Department of Respiratory and Critical Care Medicine, Zhengzhou University People’s Hospital, Henan Provincial People’s Hospital, Zhengzhou, Henan, China; Medical Center - University of Freiburg, GERMANY

## Abstract

**Background:**

Idiopathic Pulmonary Fibrosis (IPF) is a progressive and fatal lung fibrosis disease with a complex molecular mechanism that remains incompletely understood. Previous studies suggest that metabolic dysregulation, particularly cholesterol metabolism, may play an essential role in the onset and progression of IPF. However, the role of cholesterol metabolism-related genes in IPF and their relationship with prognosis have not been thoroughly explored. This study aims to systematically analyze the potential function of cholesterol metabolism-associated genes in IPF by using bulk transcriptomic data to construct a prognostic risk model and single-cell RNA sequencing data to characterize the cellular context of the model genes.

**Methods:**

Transcriptomic data (GSE150910 and GSE93606) and single-cell RNA sequencing data from IPF patients were obtained from the GEO database. Differential gene expression analysis was performed using DESeq2 and limma packages to identify significant differentially expressed genes, and a cholesterol metabolism-related gene module was constructed through WGCNA. Subsequently, key cholesterol metabolism genes associated with IPF prognosis were screened using univariate Cox regression and LASSO regression. A prognostic risk model was developed based on the selected biomarkers, and its predictive performance was evaluated using Kaplan-Meier survival curves and ROC curves. To explore the potential biological functions of these genes and their mechanisms in IPF, immune infiltration analysis, GO and KEGG functional enrichment analysis, protein-protein interaction (PPI) network analysis, and GSEA were performed. Finally, mRNA of these genes were validated in animal models.

**Results:**

A total of 19 differentially expressed cholesterol metabolism-related genes were identified from IPF patients. Through univariate Cox and LASSO regression, three key genes (*PDLIM7*, *CFAP45*, and *HP*) were selected, and a prognostic risk model for IPF patients was constructed based on these three genes. Kaplan-Meier survival analysis and ROC curves showed that the model demonstrated high predictive performance in both the training and validation cohorts (Area Under the Curve > 0.70). Additionally, immune infiltration analysis revealed significant differences in immune cell infiltration between the high-risk and low-risk groups, suggesting that cholesterol metabolism and immune regulation are closely linked to the progression of IPF. GSEA enrichment analysis highlighted pathways related to lipid metabolism and inflammatory response that were significantly enriched in the high-risk group, and animal experiments showed elevated mRNA expression of these genes in the lungs of fibrotic mice.

**Conclusion:**

This study constructed a prognostic risk model for IPF using genes derived from a cholesterol metabolism–associated module, and explored their potential roles in IPF progression.

## 1. Introduction

Idiopathic Pulmonary Fibrosis (IPF) is a progressive and life-threatening chronic interstitial lung disease of unknown etiology. It is characterized by the progressive formation of pulmonary parenchymal scars, accompanied by worsening respiratory symptoms and a decline in lung function, ultimately leading to death [1,2]. The incidence of IPF is 8.2 cases per 100,000 individuals, with its prevalence increasing with age. IPF is typically diagnosed in individuals over 50 years of age, with a higher incidence in men than in women [3,4]. Unfortunately, the median survival after diagnosis is only 2–4 years [5], and currently, lung transplantation is the only curative treatment for IPF [6]. The Food and Drug Administration has approved two antifibrotic compounds for IPF -nintedanib and pirfenidone, but these compounds only slow the progression of fibrosis rather than halting it [7]. Although several potential biomarkers for IPF have been identified, these biomarkers have not yet been translated into clinical practice. Therefore, it is crucial to identify effective biomarkers for disease severity and prognosis in IPF patients, enabling early detection of patients with poor prognosis for early intervention or referral for transplantation.

Over the past decade, metabolic dysregulation, particularly lipid metabolism disorders, has gained widespread attention due to its connection with the progression of pulmonary fibrosis. Cholesterol is a crucial component of cell membranes, involved in multiple processes such as cell signaling, membrane protein function, and cytoskeletal remodeling, playing an essential role in the structure of eukaryotic cell membranes. Cholesterol metabolism disorders can lead to the development of many diseases, including diabetes, cardiovascular diseases, and cancer [8]. Furthermore, cholesterol is involved in tumor-associated macrophage polarization and T-cell exhaustion [9–11]. Elevated cholesterol levels in the blood are associated with an increased risk of various cancers [12]. Studies have shown that cholesterol and phospholipids, by inducing endoplasmic reticulum stress, promoting cell apoptosis, and enhancing the expression of pro-fibrotic biomarkers, participate in the onset and progression of IPF [13]. However, the precise role of cholesterol metabolism-related genes (CMRGs) in IPF remains unclear.

The rapid development of single-cell RNA sequencing (scRNA-seq) technology in recent years has provided valuable insights into cellular heterogeneity and cell-to-cell communication in IPF [14]. By analyzing the gene expression profiles of individual cells, researchers can identify the specific roles of different cell types in the fibrotic process and reveal their dynamic changes. Integrating scRNA-seq data with transcriptomic data can help us understand gene regulatory networks in disease states and identify potential therapeutic targets and biomarkers. Previous studies have suggested that dysregulation of cholesterol metabolism may cause abnormal cell membrane structure and function and exacerbate fibrosis by affecting the function of macrophages, epithelial cells, and fibroblasts [15]. However, the specific molecular mechanisms of cholesterol metabolism in IPF and its relationship with prognosis remain underexplored, especially at the single-cell level.

This study is the first to construct a prognostic model for IPF based on cholesterol metabolism-related genes, systematically revealing the potential mechanisms through which these genes influence the progression of IPF. The model provides new insights into the pathogenesis of IPF and offers potential strategies for patient prognosis evaluation and targeted therapy. To further investigate the molecular mechanisms underlying IPF development, this study integrates scRNA-seq and transcriptomic data to explore the prognostic value of CMRGs in IPF, as well as their functions and potential regulatory mechanisms. This integrated approach will deepen our understanding of the pathogenesis of IPF and may pave the way for breakthroughs in treatment and prognosis evaluation for IPF patients. Specifically, this study aims to systematically explore the prognostic significance of cholesterol metabolism genes in IPF through the integration of transcriptomic and scRNA-seq data. Differential gene expression analysis of IPF patient data from the Gene Expression Omnibus (GEO) database was employed to identify candidate cholesterol metabolism-related genes. Weighted Gene Co-expression Network Analysis (WGCNA) was used to expand gene modules, and Cox and Least Absolute Shrinkage and Selection Operator (LASSO) regression models were applied to identify key genes and construct a prognostic risk model based on these genes. Additionally, Gene Ontology (GO) and Kyoto Encyclopedia of Genes and Genomes (KEGG) functional enrichment analysis, protein-protein interaction (PPI) network analysis, immune infiltration analysis. This study aims to reveal the critical regulatory role of cholesterol metabolism in the progression of IPF, providing new biomarkers for personalized treatment and offering scientific evidence for disease prognosis evaluation.

## 2. Materials and methods

### 2.1. Data sources and preprocessing

This study utilized transcriptomic and scRNA-seq datasets related to IPF from the GEO database (https://www.ncbi.nlm.nih.gov/geo/). The details are as follows:

GSE150910 (GPL24676 platform): Transcriptomic data containing 103 IPF samples and 103 normal samples, used as Training Set 1 for differential gene expression analysis.

GSE93606 (GPL11532 platform): Transcriptomic data containing 57 IPF and 20 normal samples, used as Training Set 2 for differential gene expression and WGCNA.

GSE28221 (GPL6480 platform): Transcriptomic data from 82 IPF patients was used as an independent validation set to evaluate the prognostic model’s performance.

GSE122960 (GPL20301 platform): scRNA-seq data from 8 IPF patients and 8 healthy controls used to identify and analyze key cell populations.

The expression matrix data were processed using the GEOquery R package for downloading and normalization. Gene expression levels were log2-transformed for transcriptomic data, and quality control was performed on the sample data. Additionally, the scRNA-seq dataset GSE122960 (GPL20301 platform) consisted of 8 IPF and 8 control lung tissue samples. Furthermore, 140 CMRGs were mined from MSigDB (https://www.gsea-msigdb.org/gsea/msigdb).

### 2.2. Differential expression analysis

GSE150910: Differentially expressed genes (DEGs) between 103 IPF samples and 103 normal samples were identified using the DESeq2 R package (v 3.4.1) [16] (adj. P value <0.05, |log2FC| > 0.5, correction was carried out by the Benjamini-Hochberg (BH) method). At the same time, DEGs2 in the GSE93606 dataset were ascertained via the “limma” package (v 3.54.0), and thresholds were set at adj. P value <0.05 and |log2FC| > 0.5. The two DEG sets were visualized using a volcano plot and heatmap. In detail, the volcano plot was generated with the “ggplot2” package (v 3.4.1), and the heatmap, showing the top 10 upregulated and downregulated genes ranked by log_2_FC (from high to low), was created using “ComplexHeatmap” package (v 2.14.0) [17].

### 2.3. Weighted gene co-expression network analysis (WGCNA)

In the GSE93606 dataset, the CMRGs score was calculated using the single-sample gene set enrichment analysis (ssGSEA) algorithm from the “GSVA” package (v 1.46.0) based on the expression of 140 CMRGs. IPF samples with complete survival data were divided into high and low-score groups based on the optimal cutoff of the CMRGs score. Kaplan-Meier (K-M) curves were generated using the “survminer” package (v 0.4.9) to compare survival between the groups (P < 0.05). WGCNA, implemented with the “WGCNA” package (v 1.7.1), was used to identify the module most strongly associated with the CMRGs score. First, outliers were excluded by clustering all samples in the GSE93606 dataset. The CMRGs score was incorporated, and hierarchical clustering was repeated to reclassify the samples. The optimal soft threshold (power) was determined to ensure a scale-free network topology with an R² > 0.85 and mean connectivity close to zero. A co-expression matrix was constructed with parameters: minModuleSize = 100, deepSplit = 4, and mergeCutHeight = 0.4. Distinct gene modules were identified and represented by unique colors. The correlation between the CMRGs score and each module was assessed (|cor| > 0.30, P < 0.05). The module with the strongest correlation with the CMRGs score was considered the key module, and its genes were identified as potential critical factors in cholesterol metabolism and IPF pathogenesis.

### 2.4. Identification and function analyses of candidate genes

The upregulated DEGs1 and DEGs2, as well as downregulated DEGs1 and DEGs2, were intersected to identify common DEGs in IPF. These common DEGs were further cross-referenced with the key module genes to pinpoint candidate genes. GO and KEGG analyses were performed using the “clusterProfiler” package (v 4.7.1.3) to explore the biological functions associated with the candidate genes (adj. P < 0.05, correction was carried out by the BH method). To investigate protein interactions among the candidate genes, PPI network was constructed using the STRING database (https://string-db.org/) with a confidence score > 0.4. This network was visualized using Cytoscape software (v 3.7.2).

### 2.5. Determination prognostic genes and construction of risk model

In IPF samples with complete survival data from the GSE93606 dataset, univariate Cox regression analysis (HR ≠ 1, P < 0.05) and PH assumption test (P > 0.05) were employed to identify survival-related genes. Specifically, univariate Cox regression analysis was carried out via the “survival” package (v 3.3−1) [18], while the PH assumption test was conducted using the cox.zph function in R software (v 4.2.3). A forest plot of the survival-related genes was then generated by the “forestplot” package (v 2.0.1) [https://CRAN.R-project.org/package=forestplot]. Subsequently, LASSO method was applied to identify prognostic genes (family = Cox) by “glmnet” package (v 4.1.4) [19], with 10-fold cross-validation employed to ascertain the optimal lambda value (lambda min). Following this, the expression status of prognostic genes in GSE93606 was analyzed utilizing Wilcoxon test (P < 0.05). A risk model was constructed relying on the identified prognostic genes, with the risk score calculated as follows:


$$risk\;score=\sum_{i=\text{1}}^{n}\left(coefi*Xi\right)$$

where “coef” represented the coefficients of the prognostic genes in LASSO analysis and “expression” indicated their expression levels.

After that, to evaluate the properties of this risk model, 57 IPF patients from the GSE93606 dataset were stratified into high-risk (HRG) and low-risk (LRG) groups relying on the median risk score. Risk curves were generated to show the distribution of risk scores, and scatter plots were created to depict survival states for both HRG and LRG. K-M survival curves were plotted using the “survminer” package (v 0.4.9) to assess survival differences between the two groups (P < 0.05). Additionally, 1-, 2-, and 3-year survival rate ROC curves were built by “survivalROC” package (v 1.0.3) (Area Under the Curve (AUC) > 0.70). An expression heatmap of the prognostic genes was generated for both HRG and LRG, and the correlation between these prognostic genes was analyzed.

To validate the risk model’s robustness, the same analyses were applied to the GSE28221 dataset, which included 45 IPF samples with complete survival information, following the same methods and steps.

### 2.6. Gene set enrichment analysis (GSEA)

In the GSE93606 dataset, enrichment analysis was conducted to further investigate the biological function differences between the HRG and LRG. First, differential expression analysis (HRG vs LRG) was conducted, and obtained genes were ranked based on their log2 FC values, from highest to lowest. GSEA was then carried out using the “clusterProfiler” package (v 4.7.1.3) (adj. P < 0.05, correction was carried out by the BH method). The parameters were set as follows: species = “Homo sapiens”, category = “C2”, and subcategory = “CP:KEGG”.

### 2.7. Immune infiltration analysis

To investigate immune cell infiltration in IPF samples, an immune infiltration analysis was applied to the GSE93606 dataset. CIBERSORT algorithm (v 1.03) was first used to calculate the infiltration proportions for 22 immune cell types. Cells that had a value of 0 in 30% of the samples were excluded from further analysis. The remaining cells were considered as infiltrating cells. Wilcoxon test was then applied to compare infiltration proportions between the HRG and LRG, identifying immune cells with significant differences (P < 0.05) for further investigation. Subsequently, Spearman correlation analysis was performed by “corrplot” package (v 0.92) to explore the relationships between infiltrating cells (|cor| > 0.3 and P < 0.05), as well as between prognostic genes and differential immune cells (|cor| > 0.3 and adj.P < 0.05, correction was carried out by the BH method). To further validate the results of the immune infiltration analysis, the ssGSEA algorithm from the “GSVA” package (v 1.46.0) was used to calculate the proportions of 28 immune cell types in disease samples from the GSE93606 dataset. The Wilcoxon test was applied to compare the differences in immune cell infiltration proportions between HRG and LRG samples (P < 0.05).

### 2.8. Regulation network analysis and compound prediction analysis

The miRNA network (miRNet, https://www.mirnet.ca) database predicted microRNAs (miRNAs) targeting the prognostic genes. This database was also employed to predict long non-coding RNAs (lncRNAs) targeting the identified miRNAs. The relationships between lncRNAs, miRNAs, and mRNAs were then organized to construct and visualize a lncRNA-miRNA-mRNA network using Cytoscape (v 3.7.2). Additionally, the compound Signatures Database (DSigDB) (http://tanlab.ucdenver.edu/DSigDB) was utilized to predict compounds targeting the prognostic genes. A biomarker-compound interaction network was subsequently built and visualized using Cytoscape (v 3.7.2).

### 2.9. Single-cell analysis

The core statistical unit in the single-cell analysis of this study is the “donor”. The scRNA-seq data from GSE122960 were analyzed using the “Seurat” package (v 5.0.1) [20]. Quality control (QC) filtered out low-quality cells and genes, with the following criteria: genes expressed in fewer than 3 cells were excluded, and retained cells had 200 < nFeature-RNA < 6,000, nCount-RNA < 20,000, and percent.mt < 10%. Data were then normalized using the NormalizeData function, and highly variable genes (HVGs) were identified with the FindVariableFeatures function (selection.method = “vst”). Principal component analysis (PCA) was performed on HVGs using the RunPCA function, and principal components (PCs) were selected with the JackStraw function (P < 0.05). Uniform manifold approximation and projection (UMAP) dimensionality reduction was conducted at a resolution of 0.4, followed by cell clustering using FindNeighbors and FindClusters. Cell types were annotated using the cellMarker function and the “singleR” package (v 2.0.0), and visualized with UMAP.

To identify key cells associated with IPF, the Wilcoxon test was used to compare the proportion of annotated cells between IPF and control samples. Cells with significant differences and higher proportions in IPF and control samples were considered key cells. The expression of prognostic genes in each annotated cell was analyzed between IPF and control samples (P < 0.05).

### 2.10. Cell-to-cell communication analysis and pseudo-temporal analysis

To gain a deeper understanding of the interactions between key cells and other annotated cell clusters in the GSE122960 dataset, cell-to-cell communication analysis was carried out by CellChat package (v 1.6.1) [21]. Following this, CellPhoneDB v2.0 (https://www.cellphonedb.org/), a signaling molecule interaction database, was employed to predict the enriched signals and interactions between cell clusters. This analysis assessed the expression levels of receptors and ligands and inferred potential intercellular interactions. Finally, to explore the differentiation states and trajectories of key cells, pseudo-temporal analysis was conducted using the “Monocle” package (v 2.22.0) [22]. The expression changes of prognostic genes throughout the pseudo-temporal progression were also visualized.

### 2.11. Animals

Six- to eight-week-old C57BL/6J mice (n = 12) were obtained from the Experimental Animal Center of Zhengzhou University. All animal procedures were approved by the Ethics Committee of Zhengzhou University (ZZU-LAC20230526[01]) and were reported in accordance with ARRIVE guidelines (https://arriveguidelines.org). Current sample size (n = 6 per group) was based on prior experience. Mice were randomly assigned to two groups using a computer-generated randomization list (n = 6/group): control (Ctrl) and bleomycin (BLM). Pulmonary fibrosis was induced by intratracheal administration of bleomycin (2.5 mg/kg) using a mouse intubation kit (Ruiwode Inc., Shenzhen, China) under anesthesia with intraperitoneal pentobarbital sodium (50 mg/kg). Control mice received an equal volume of sterile saline. Post-procedural analgesia was provided with buprenorphine (0.1 mg/kg, subcutaneously, every 12 h for 48 h). Mice were monitored daily, and predefined humane endpoints included ≥20% body weight loss, severe respiratory distress, inability to access food/water, or persistent recumbency; mice meeting endpoints were euthanized by pentobarbital overdose. On day 21 after treatment, lungs were collected for histology and hydroxyproline measurement. Fibrosis severity was quantified using Ashcroft scoring on 10 randomly selected non-overlapping fields per mouse at 20 × magnification by two blinded assessors, with the mean score per mouse used for analysis.

### 2.12. Hydroxyproline analysis

Hydroxyproline content was measured using a commercial kit (A030-2–1, Njjccbio, China). Lung tissue was homogenized in 0.9% saline (weight (mg):volume (µL) = 1:9), centrifuged at 690 × g for 10 min, and the supernatant was assayed according to the manufacturer’s protocol.

### 2.13. CT imaging

Pulmonary fibrosis in mice was assessed using micro-CT imaging. Mice were anesthetized with isoflurane via a nasal mask and positioned in the prone position on the imaging bed. CT scans were performed using a micro-CT-NEMO machine (NMC-200, PingSheng Medical Technology, Inc., China) with GPU acceleration and respiratory gating. Images were acquired at a spatial resolution of 7.5 μm (10% MTF) with 720 views per mouse. Data acquisition, reconstruction, and analysis were done using Crassier, Recon, and Avatar software. Three mice per group were scanned.

### 2.14. Hematoxylin and eosin (H&E)

As previously described [23], Mouse lung tissue was fixed in 4% formalin overnight, embedded in paraffin, and sectioned into 5 μm slices using a microtome. After dewaxing and rehydration, sections were stained with hematoxylin and eosin (G1003, Servicebio, China) for 5 minutes, following standard protocols. Tissue sections were examined under a light microscope, and the extent of fibrosis was assessed using the Ashcroft score for H&E-stained sections [24].

### 2.15. Reverse transcription‑quantitative polymerase chain reaction (RT qPCR)

Total RNA was extracted from mouse lung tissue using RNA extraction solution (G3013, Servicebio, China) and its concentration was measured with a UV spectrophotometer (NANO 2000). First-strand cDNA was synthesized from the RNA using a cDNA synthesis kit, and the resulting cDNA was used as a template for quantitative PCR. The primers, cDNA, and reagents were mixed according to the manufacturer’s protocol, and quantitative fluorescence analysis was performed using an ExicyclerTM 96 (Bioneer, Korea). The fold change in mRNA expression was calculated using the ΔΔCt method, with GAPDH as a control. RT-qPCR was used to measure the levels of PDZ and LIM Domain 7 (*PDLIM7*), Cilia and Flagella Associated Protein 45 (*CFAP45*), and Haptoglobin (*HP*). The primers used in this study are listed in S1 Fig.

### 2.16. Statistical analysis

Statistical analysis was performed using R (v 4.2.3). The Wilcoxon test (P < 0.05) assessed the two groups’ differences. Animal data are presented as mean ± standard deviation (SD) and were obtained from at least three independent experiments. T-test was used to evaluate statistical differences among animal samples, with significance set at P < 0.05. GraphPad Prism 8.0 software was used for data analysis and graph generation.

## 3. Results

### 3.1. Identification of DEGs and key module genes

**Fig 1:**
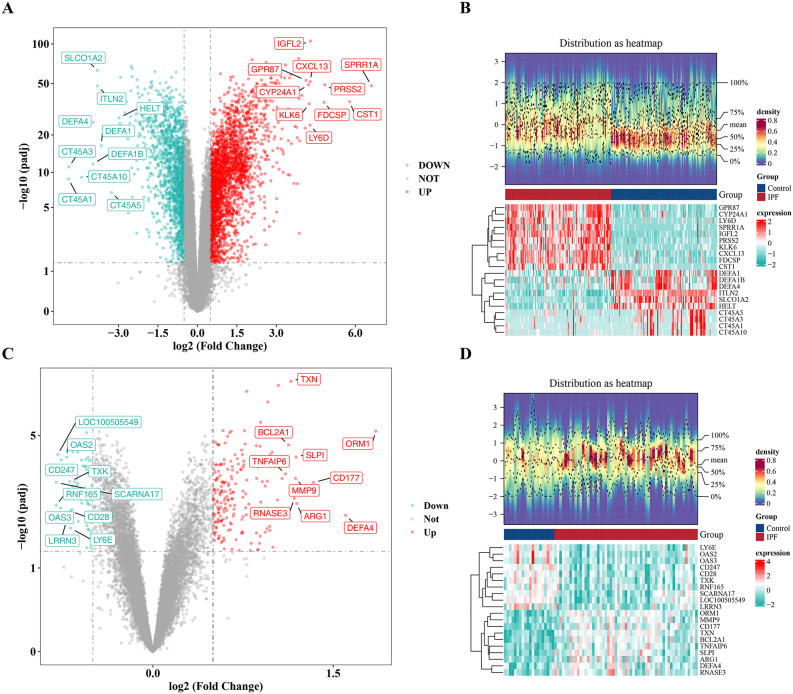
Identification of Differentially Expressed Genes (DEGs). **A.** Volcano plot of DEGs in the GSE150910 dataset (103 IPF samples vs 103 normal samples). **B.** Heatmap of DEGs in the GSE150910 dataset (the upper part shows a density heatmap of DEG expression across samples, displaying five quantiles and mean lines; the lower part shows a heatmap of DEG expression). **C.** Volcano plot of DEGs in the GSE93606 dataset (57 IPF vs 20 normal samples). **D.** Heatmap of DEGs in the GSE93606 dataset (similar to panel **B)**. Red dots represent upregulated genes, green dots represent downregulated genes, and gray dots represent non-differentially expressed genes.

In the GSE150910 dataset, 4,216 DEGs1 were identified, with 2,632 up- and 1,584 down-regulated DEGs1 in IPF samples (Fig 1A-B). In the GSE93606 dataset, 257 DEGs2 were ascertained, including 190 up- and 67 down-regulated DEGs2 in IPF samples (Fig 1C-D).

**Fig 2:**
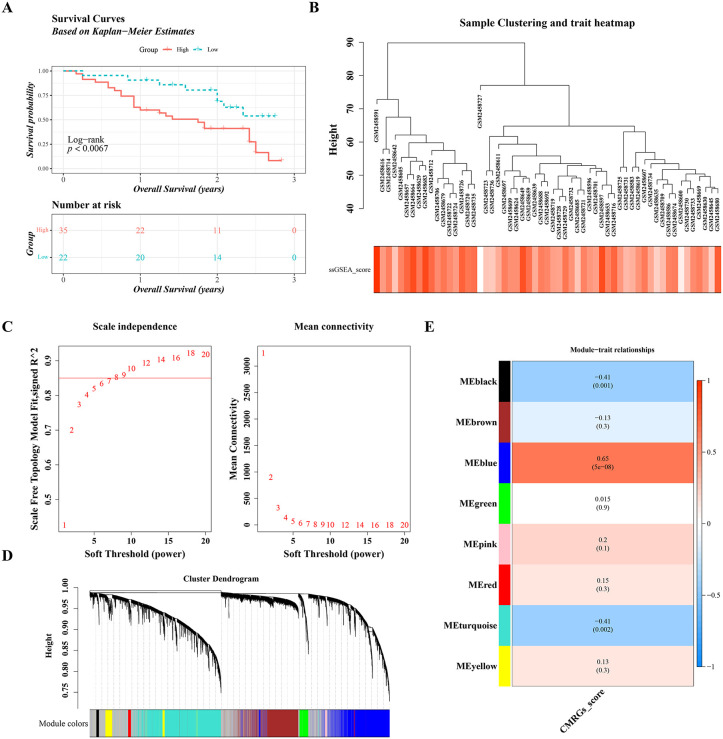
Identification of Key Module Genes. **A.** Kaplan-Meier survival curves for IPF samples (Log-rank test). **B.** Hierarchical clustering of samples (each branch represents a sample; the vertical axis indicates the Euclidean distance of gene expression levels). **C.** Soft-threshold selection (the left plot suggests 9 as the optimal soft-threshold for further analysis; the right plot shows network connectivity under different soft-thresholds). **D.** Identification of co-expression modules (the upper part shows a hierarchical clustering dendrogram of genes, and the lower part represents gene modules). **E.** Heatmap of module-trait correlations (left color blocks represent modules, right color bar represents the correlation range; in the middle heatmap, darker colors indicate higher correlations, red represents positive correlation, blue represents negative correlation, and cell numbers denote correlation and significance).

Among the 57 IPF samples with available survival data in GSE93606, the samples were divided into a high-score group (35 samples) and a low-score group (22 samples). K-M curve revealed that the low-score group had an obviously higher survival probability (P < 0.0067) (Fig 2A). For WGCNA, no outlier samples were detected (Fig 2B). The optimal power value was determined to be 9, as this produced an R² value greater than 0.85 (indicated by the red line) and resulted in mean connectivity close to 0 (Fig 2C). The co-expression matrix was then used to merge similar modules, leading to the identification of 8 gene modules, excluding the gray module, which represents unclassified genes (Fig 2D). The Meblue module (cor = 0.65, P = 5 × 10 ⁻ ⁸) showed a strong association with the CMRGs score (Fig 2E). As a result, the 3,792 genes within the Meblue module were identified as key module genes, providing a foundation for subsequent gene screening.

### 3.2. Ascertainment and functional exploration of candidate genes

**Fig 3:**
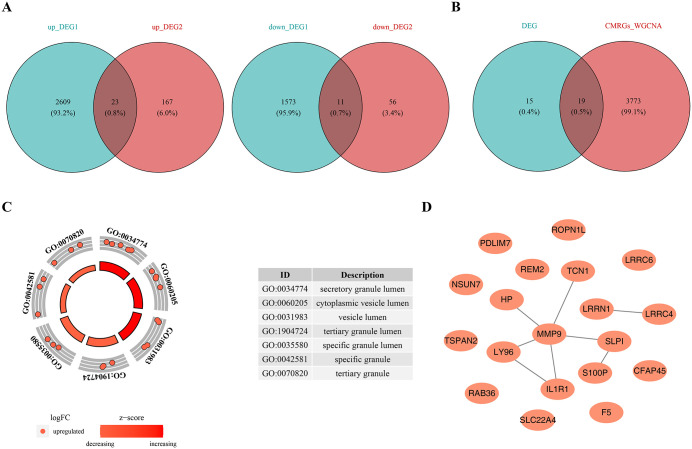
Ascertainment and Functional Exploration of Candidate Genes. **A.** Intersection of IPF DEGs (left: upregulated genes, right: downregulated genes). **B.** Differentially expressed CMRGs. **C.** GO enrichment analysis of candidate genes. **D.** Protein-protein interaction (PPI) network of candidate genes.

The up-regulated DEGs1 and DEGs2 overlapped to identify 23 genes, while the down-regulated DEGs1 and DEGs2 overlapped to reveal 11 genes, resulting in a total of 34 common DEGs (Fig 3A). Subsequently, 19 candidate genes were identified by intersecting these 34 common DEGs with 3,792 key module genes (Fig 3B). Functional enrichment analysis revealed significant associations with 7 GO terms, all related to cellular components (CCs), such as “secretory granule lumen” and “cytoplasmic vesicle lumen” (Fig 3C). Unfortunately, the candidate genes were not significantly enriched in KEGG pathways. Additionally, a PPI network was constructed for the 19 candidate genes at a confidence level of 0.4, revealing 8 interactions (Fig 3D). Notably, MMP9 showed significant interactions with several genes, including IL1R1 and LY96, within this network.

### 3.3. Acquisition of the prognostic genes

**Fig 4:**
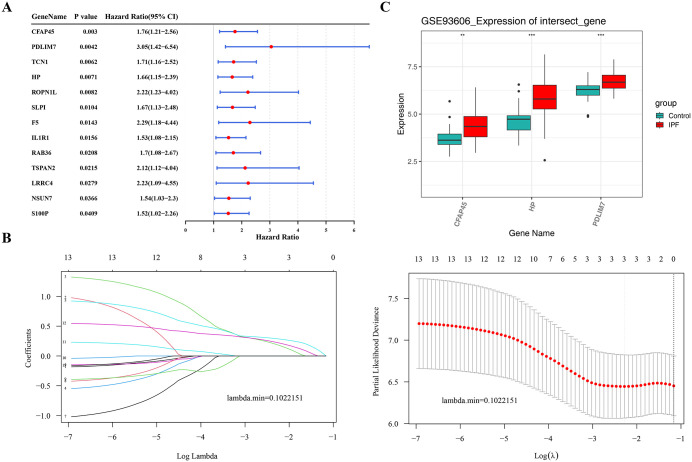
Acquisition of Prognostic Genes. **A.** Forest plot of univariate Cox regression analysis. **B.** LASSO regression analysis. **C.** Expression levels of biomarkers in IPF samples and controls (Wilcoxon test). ** represents *P* < 0.01, and *** represents *P* < 0.001.

From an initial set of 19 candidate genes, 13 survival-related genes were ascertained through regression analysis (Fig 4A) and the PH assumption test (S2 Fig). Notably, these 13 survival-related genes were found to be risk factors for IPF, with HR > 1 and P < 0.05. Following this, the LASSO method further narrowed down the list of 3 prognostic genes (*PDLIM7*, *CFAP45*, and *HP*) based on a lambda min of 0.102 and non-zero regression coefficients (Fig 4B). Interestingly, the expression of all 3 prognostic genes were obviously higher in IPF samples (P < 0.05) (Fig 4C). These findings highlighted the potential role of *PDLIM7*, *CFAP45*, and *HP* in the treatment and prognosis of IPF.

### 3.4. Construction of well-performing risk model

**Fig 5:**
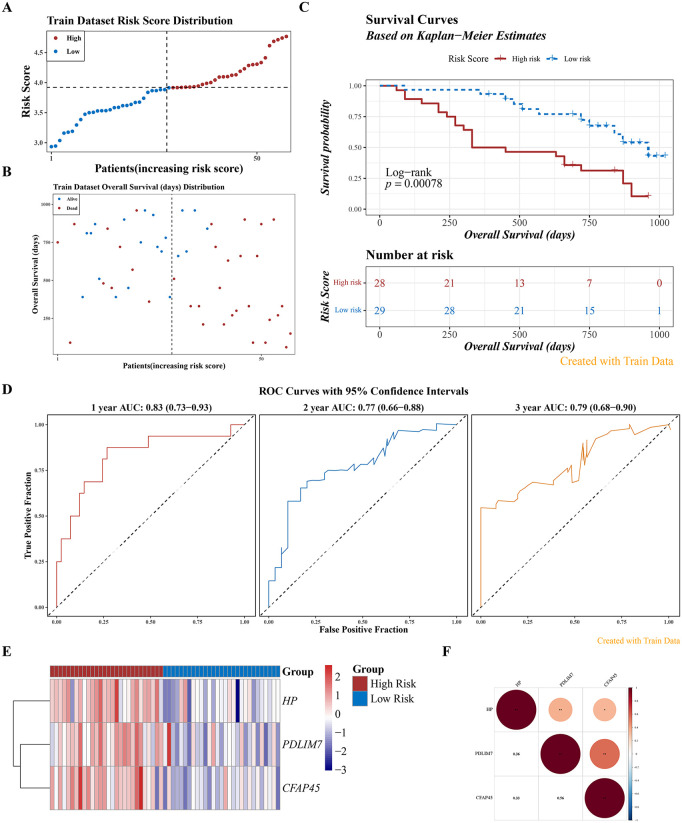
Construction of a Well-Performing Risk Model. **A.** Risk score distribution (x-axis: samples sorted by increasing risk score; y-axis: risk score; dashed lines indicate median risk score and corresponding patient count). **B.** Survival status distribution (x-axis: samples sorted by increasing risk score; y-axis: survival time; dashed lines indicate median risk score and corresponding patient count). **C.** Kaplan-Meier survival curve (Log-rank test). **D.** ROC curve (nodes at 1, 2, and 3 years). **E.** Heatmap showing *HP*, *PDLIM7*, and *CFAP45* expression levels in high-risk group (n = 28) and low-risk group (n = 29). **F.** Correlation among the three model genes.

**Fig 6:**
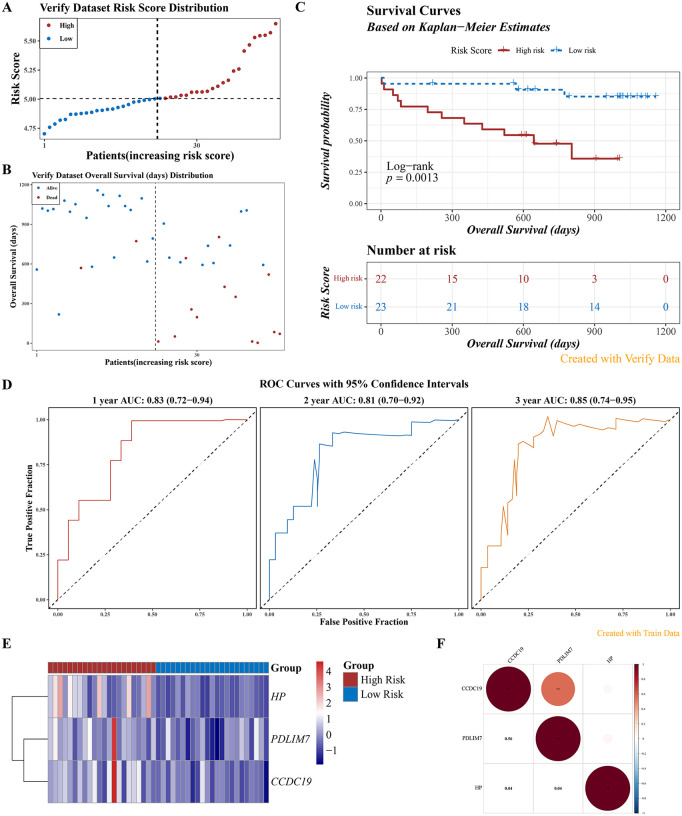
Validation of the Well-Performing Risk Model. **A.** Risk score distribution (x-axis: samples sorted by increasing risk score; y-axis: risk score; dashed lines indicate median risk score and corresponding patient count). **B.** Survival status distribution (x-axis: samples sorted by increasing risk score; y-axis: survival time; dashed lines indicate median risk score and corresponding patient count). **C.** Kaplan-Meier survival curve (Log-rank test). **D.** ROC curve (nodes at 1, 2, and 3 years). **E.** Heatmap showing *HP*, *PDLIM7*, and *CFAP45* expression levels in high-risk group (n = 22) and low-risk (n = 23) group. **F.** Correlation among the three model genes.

A risk model was developed based on 3 prognostic genes: Risk Score = 0.191 × *PDLIM7* + 0.290 × *CFAP45* + 0.227 × *HP*. This model successfully stratified 57 IPF patients from the GSE93606 dataset into 2 groups: HRG (28 samples) and LRG (LRG, 29 samples). Similarly, 45 IPF patients from the GSE28221 dataset were classified into HRG (22 samples) and LRG (23 samples). In the GSE93606 dataset, risk curves and survival status distributions differentiated the 2 groups, with HRG patients exhibiting shorter survival times (Fig 5A-B). K-M survival curves further revealed notably lower survival probabilities for HRG patients (P < 0.01) (Fig 5C). ROC curves demonstrated strong predictive performance, with all AUC values exceeding 0.70 in the GSE93606 dataset (Fig 5D). Moreover, the expression levels of the 3 prognostic genes, *PDLIM7*, *HP*, and *CFAP45*, were relatively higher in HRG patients (Fig 5E). Correlation analysis showed a strong positive correlation between *PDLIM7* and *CFAP45* (cor > 0.5, P < 0.05) (Fig 5F). These findings were consistently validated in the GSE28221 dataset (Fig 6A-F). In conclusion, the developed risk model effectively stratified IPF patients, offering promising potential for personalized treatment strategies and improved disease management.

### 3.5. Exploration of pathway and immune infiltration differences in HRG and LRG patients

**Fig 7:**
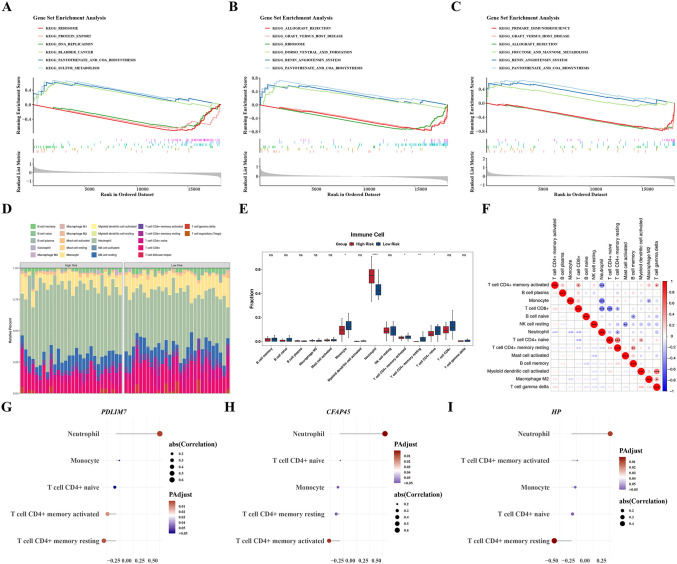
Exploration of Pathway and Immune Infiltration Differences Between HRG and LRG Patients. **A-C.** Functional enrichment analysis of *PDLIM7*, *CFAP45*, and *HP*, respectively. **D.** Proportion of 22 immune cell types. **E.** Boxplots of 14 immune cell score differences (Wilcoxon test). The horizontal axis represents different types of immune cells, and the vertical axis represents the proportion of cell infiltration. ns indicates *P* > 0.05, * indicates *P* < 0.05, ** indicates *P* < 0.01, and **** indicates P < 0.0001. **F.** Correlation heatmap of 14 immune cell types. Red indicates a positive correlation, and blue indicates a negative correlation. The darker the color, the stronger the correlation. **G-I.** Lollipop charts showing *PDLIM7*, *CFAP45*, and *HP* correlations with differential immune cells, respectively.

In the GSEA analysis of *PDLIM7*, pathways such as “bladder cancer”, “pantothenate and CoA biosynthesis”, and “sulfur metabolism” were significantly enriched in the HRG, while pathways like “ribosome”, “protein export”, and “DNA replication” were notably enriched in the LRG (Fig 7A). *CFAP45* showed “dorso-ventral axis formation”, “renin-angiotensin system”, and “pantothenate and CoA biosynthesis” were obviously enriched in HRG, while pathways for instance “ribosome”, “allograft rejection”, and “graft-versus-host disease” were notably enriched in LRG (Fig 7B). For *HP*, pathways like “fructose and mannose metabolism”, “renin-angiotensin system”, and “pantothenate and CoA biosynthesis” were prominently enriched in HRG, whereas pathways such as “primary immunodeficiency”, “graft-versus-host disease”, and “allograft rejection” were notably enriched in LRG (Fig 7C). Overall, these results suggested that *PDLIM7*, *CFAP45*, and *HP* played essential roles in regulating key metabolic and immune-related pathways.

Moreover, the heatmap illustrated the immune infiltration status of 14 infiltrating cell types in IPF samples (Fig 7D). Obvious differences in the infiltration levels of 5 immune cells were observed (HRG vs LRG) (P < 0.05) (Fig 7E). For instance, monocytes and naïve CD4 + T cells were more prevalent in LRG samples. At the same time, neutrophils showed higher infiltration in HRG patients. Further analysis revealed a strong negative correlation between neutrophils and monocytes (cor = −0.55, P < 0.05). Additionally, resting memory CD4 + T cells were positively correlated with naïve CD4 + T cells (cor = 0.53, P < 0.05) (Fig 7F). Correlation between prognostic genes and differential immune cells showed that neutrophils had a significant positive correlation with *PDLIM7* (cor = 0.63, adj.P < 0.05) and *CFAP45* (cor = 0.67, adj.P < 0.05). Furthermore, *HP* was negatively correlated with resting memory CD4 + T cells (cor = −0.46, adj.P < 0.05) (Fig 7G-I). In addition, the ssGSEA algorithm showed that there were 13 differentially infiltrated immune cell types between HRG and LRG, such as neutrophils, central memory CD4 + T cells, and effector memory CD4 + T cells. These results were similar to those obtained with the CIBERSORT algorithm, which indicated the reliability of the immune infiltration results. In summary, the analysis revealed distinct immune cell infiltration patterns associated with risk levels in IPF patients, which could provide valuable insights for developing targeted immunotherapies for IPF.

### 3.6. Revealing potential regulatory mechanisms and potential therapeutic compounds of IPF

**Fig 8:**
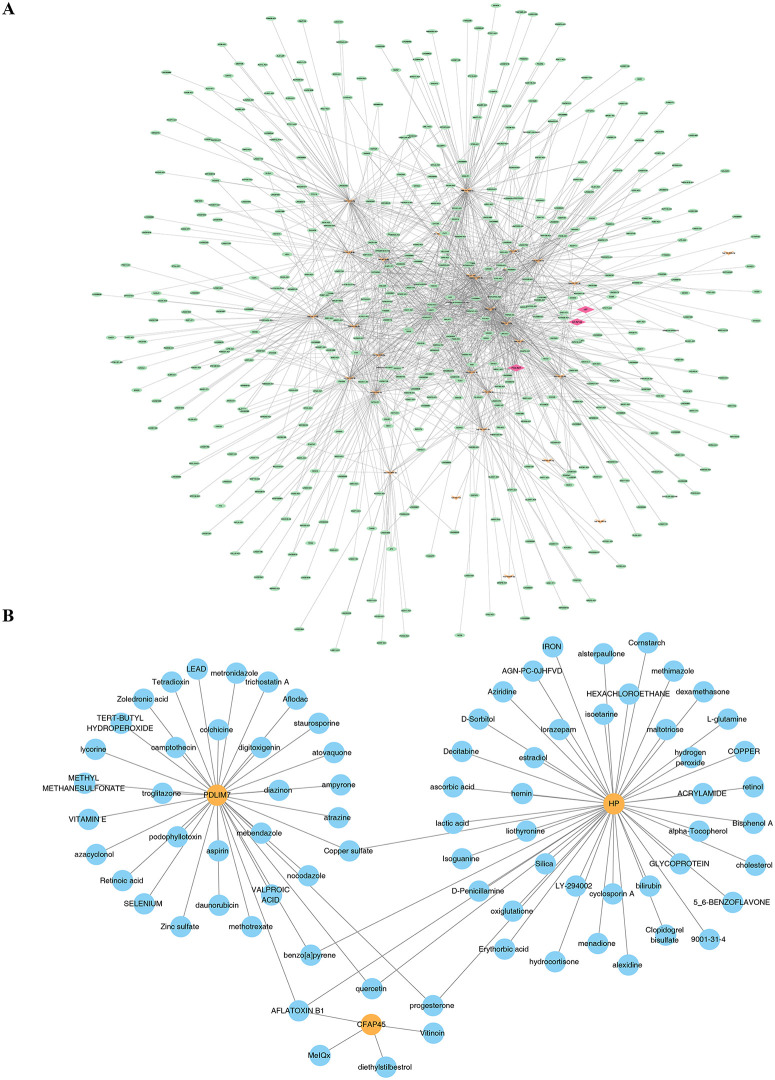
Revealing Potential Regulatory Mechanisms and Therapeutic compounds for IPF. **A.** Construction of the ceRNA network (pink nodes represent mRNA biomarkers, green nodes represent lncRNAs, and orange nodes represent miRNAs). **B.** Biomarker-small molecule compound network (red nodes represent mRNA biomarkers, green nodes represent small molecule compounds).

37 miRNAs were identified that targeted prognostic genes, including 23 miRNAs targeting *PDLIM7*, 8 miRNAs targeting *CFAP45*, and 6 miRNAs targeting *HP*. Furthermore, 1,514 miRNA-lncRNA relationships were explored, leading to the construction of a regulatory network. For instance, the miRNAs included hsa-miR-124-3p, hsa-miR-342-3p, and hsa-miR-16-5p, while notable miRNA-lncRNA relationships included hsa-miR-124-3p-PCGEM1, hsa-miR-342-3p-XIST, and others (Fig 8A). Additionally, 35 compounds targeting *PDLIM7*, 4 compounds targeting *CFAP45*, and 47 compounds targeting *HP* were predicted (Fig 8B). These findings provided valuable insights into the potential application of targeted miRNAs and compounds in regulating prognosis genes, offering promising directions for future research in treatment and personalized medicine.

### 3.7. Identification of epithelial cells and macrophages as key cells for IPF

**Fig 9:**
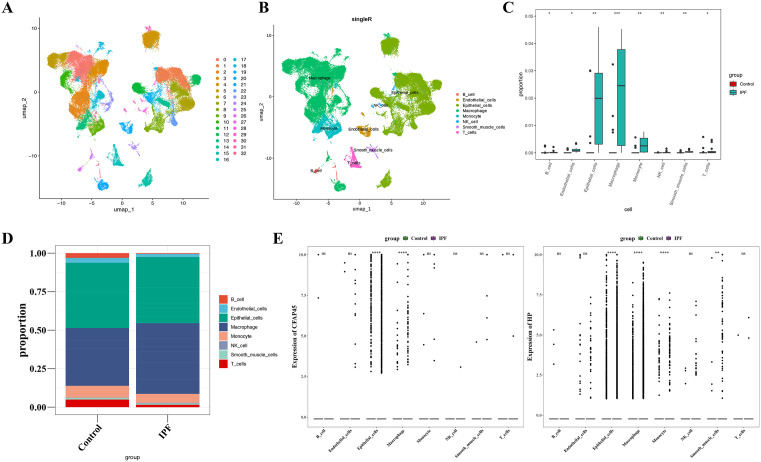
Identification of Epithelial Cells and Macrophages as Key Cells for IPF. **A.** Dimensionality-reduced cell clusters. **B.** Annotated cell clusters. **C.** Differences in cell types between IPF and normal samples (Wilcoxon test). **D.** Proportions of differential cell types. **E.** Expression levels of biomarkers in different cell types (Wilcoxon test). * indicates *P* < 0.05, ** indicates *P* < 0.01, and *** represents *P* < 0.001.

Initially, 72,909 cells and 33,694 genes were retained after removing ineligible cells and genes (S4A Fig). Next, 2,000 HVGs were identified (S4B Fig), and PCA was performed, selecting the top 30 PCs for further analysis (P < 0.05) (S4C-4D Fig). Using UMAP analysis, the cells were classified into 33 distinct clusters (Fig 9A). Among these, 8 clusters were annotated as B cells, endothelial cells, epithelial cells, macrophages, monocytes, natural killer (NK) cells, T cells, and smooth muscle cells (Fig 9B). The proportion of these 8 cell types showed significant differences between IPF and control samples (P < 0.05) (Fig 9C). Notably, epithelial cells and macrophages exhibited a significantly higher proportion in both IPF and control samples, suggesting they play key roles in IPF (Fig 9D). Therefore, epithelial cells and macrophages were identified as key cell types in IPF. Additionally, within epithelial cells and macrophages, the expression of *CFAP45* showed significant differences between IPF and control samples (P < 0.05). In contrast, in epithelial cells, macrophages, monocytes, and smooth muscle cells, the expression of *HP* exhibited significant differences between IPF and control samples (Fig 9E). Regretfully, this analysis was conducted based on HVGs. As a result, *PDLIM7* was not identified, with only *CFAP45* and *HP* being highlighted.

Overall, these findings highlighted the differential distribution and expression profiles of key cell types, particularly epithelial cells and macrophages, in the context of IPF.

### 3.8. Exploration interactions and differentiation trajectories of key cells

**Fig 10:**
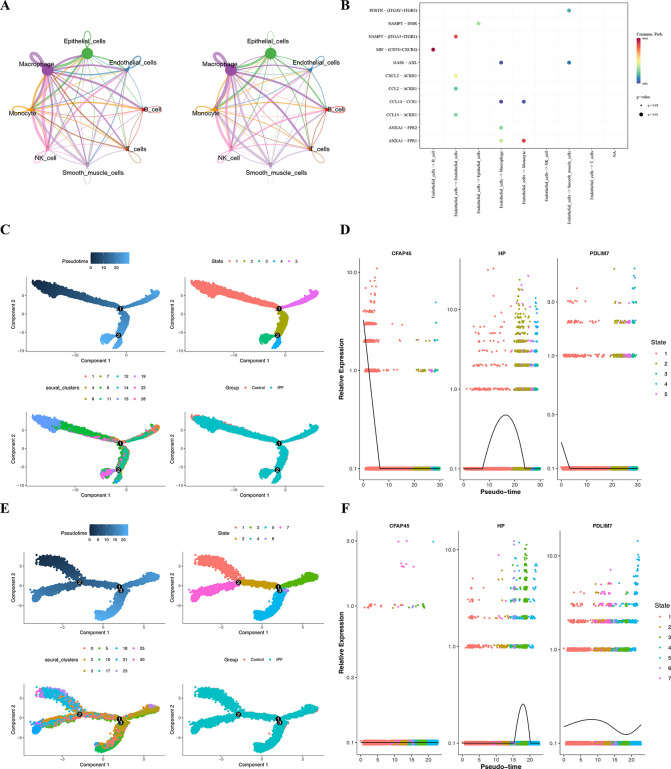
Exploration of Interactions and Differentiation Trajectories of Key Cells. **A.** Cell-cell communication networks among eight cell types (left: by interaction count, right: by weight; different colors represent different cell types, line thickness indicates interaction strength). **B.** Bubble chart of receptor-ligand interactions between cells (bubble size represents p-value; red indicates high communication probability, blue indicates low communication probability). **C-D.** Pseudotime trajectory analysis and relative expression profiles of biomarkers in epithelial cells (x-axis: pseudotime, darker colors represent earlier pseudotime, lighter colors represent later pseudotime). **E-F.** Pseudotime trajectory analysis and relative expression profiles of biomarkers in macrophages.

Cell-to-cell communication analysis revealed frequent interactions between macrophages, epithelial cells, and monocytes (Fig 10A, S5 Fig). Additionally, monocytes and macrophages interacted through the LGALS9-CD44 (Fig 10B). These findings suggested dynamic interactions between key cells and monocytes in the context of IPF.

Moreover, pseudo-temporal analysis showed that the differentiation trajectory of epithelial cells progressed from left (dark blue) to right (light blue), with cells being distinctly classified into 5 states (Fig 10C). State 1 represented the earliest differentiation stage. In epithelial cells, the expression of the prognostic genes *CFAP45* and *PDLIM7* decreased over time, while *HP* expression initially increased and then decreased (Fig 10D).

Similarly, the differentiation trajectory of macrophages progressed from left (dark blue) to right (light blue), with cells classified into 7 distinct states (Fig 10E). State 1 represented the earliest stage of differentiation. In macrophages, *HP* expression initially increased and then decreased, *PDLIM7* expression first increased, then decreased, and later rose again. In contrast, *CFAP45* expression showed no apparent changes (Fig 10F). Overall, the differentiation trajectories of both epithelial cells and macrophages reveal temporal changes in the expression of prognostic genes, *PDLIM7*, *CFAP45*, and *HP*, which might reflect critical biological processes in the disease progression.

### 3.9. Biomarker Expression Validation in mice

**Fig 11:**
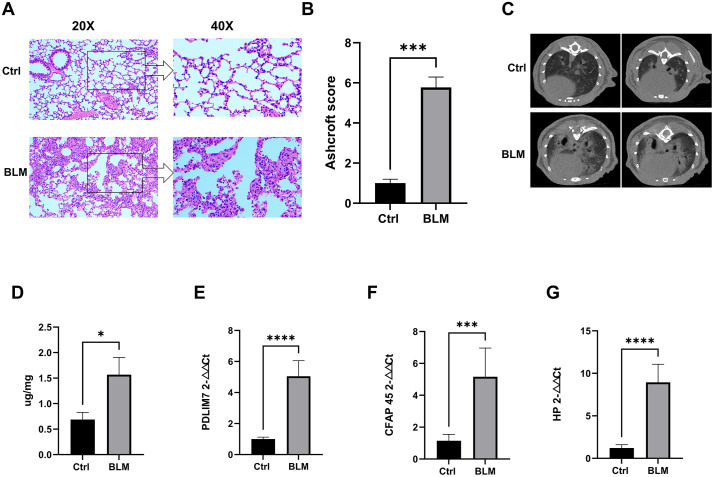
Validation of Biomarker Expression in Mice. **A.** H&E staining of lung tissues (scale bar: 100 μm). **B.** Quantification of pulmonary fibrosis severity using the **Ashcroft score** based on **H&E-stained** mouse lung sections. **C.** Chest CT images of mice. **D.** Lung hydroxyproline content measured using a commercial hydroxyproline assay kit (A030-2-1, Njjccbio, China). **E-G.** mRNA levels of *PDLIM7*, *CFAP45*, and *HP* in lung tissues determined by RT-qPCR (6 Ctrl vs 6 BLM). GAPDH was used as the housekeeping gene for normalization. Results are expressed as relative gene transcript levels, with the Ctrl group set to 1. Data are presented as mean ± SD. Error bars represents SD. * *P* < 0.05; **P < 0.01; ***P < 0.001.

The *PDLIM7*, *HP*, and *CFAP45* genes, in particular the *HP* gene, had high homologies between humans and mice with little difference. To further assess the reproducibility of these biomarkers, we employed a bleomycin-induced mouse model of pulmonary fibrosis [23]. Both control and experimental groups were established, and the successful induction of pulmonary fibrosis was verified through HE staining (Fig 11A), Ashcroft score (Fig 11B) and CT imaging of the mouse lungs (Fig 11C),and hydroxyproline content (Fig 11D). Subsequently, qRT-PCR analysis was conducted to measure the expression levels of *PDLIM7* (Fig 11E), *CFAP45* (Fig 11F), and *HP* (Fig 11G). The results showed a significant increase in the expression of these biomarkers in the lungs of bleomycin-treated mice compared to the control group, supporting their association with fibrotic lung remodeling.

## 4. Discussion

IPF is a severe and progressive fibrotic lung disease characterized by gradual respiratory deterioration and impaired lung function [25]. The exact mechanisms underlying IPF remain unclear, and current therapeutic options do not significantly slow disease progression or improve long-term prognosis [26]. Consequently, identifying novel biomarkers that can better predict disease progression and enable personalized therapeutic strategies is a critical research focus. This study integrated transcriptomic and scRNA-seq data to explore the prognostic value of cholesterol metabolism-related genes in IPF. We successfully constructed a prognostic model based on cholesterol metabolism-related genes through comprehensive bioinformatic analyses and identified three key biomarkers: *PDLIM7*, *CFAP45*, and *HP*. Our findings suggest that cholesterol metabolism may play a significant role in the onset and progression of IPF. The integration of single-cell sequencing revealed that these genes might influence patient outcomes by modulating fibrosis-related cells, such as epithelial cells and macrophages, and immune responses. These results provide novel insights into IPF pathogenesis and strong support for personalized treatment and prognosis assessment in IPF.

Cholesterol metabolism is critical in cellular structure, signaling, and immune responses. Recent studies have shown that dysregulated cholesterol metabolism is closely associated with several chronic lung diseases, such as chronic obstructive pulmonary disease and asthma [27–29]. For the first time, this study links cholesterol metabolism to the pathological progression of IPF. Among the 19 identified CMRGs, GO and KEGG enrichment analyses revealed their significant involvement in cellular components such as cytoplasmic vesicles and specific granule lumens. These results suggest that cholesterol metabolism-related genes may influence key pathological mechanisms in IPF by regulating cell membrane structure and vesicular transport. Cholesterol metabolism is essential not only for maintaining cellular membrane integrity and vesicle formation but also for modulating cellular signaling pathways involved in fibrosis progression. For instance, cholesterol and its derivatives serve as signaling molecules within membranes, regulating fibroblast migration, proliferation, and fibrogenesis [13]. Additionally, the relationship between cholesterol metabolism and inflammatory responses has garnered increasing attention. Studies indicate that dysregulated cholesterol metabolism may enhance the pro-fibrotic functions of macrophages and monocytes by affecting their activation [30]。

*PDLIM7*, *CFAP45*, and *HP* were identified as the key genes for constructing the prognostic model through univariate Cox regression and LASSO regression. These genes were highly expressed in IPF patients and closely correlated with patient survival, suggesting their significant roles in IPF progression. These aberrantly expressed genes have also been confirmed in animal studies.

*PDLIM7* is a member of the PDZ and LIM domain protein family, which regulates cytoskeletal remodeling and cell motility through interactions with cytoskeleton-associated proteins. As a regulator of actin-rich membrane protrusions, *PDLIM7* promotes cellular motility and invasiveness by enhancing cytoskeletal remodeling [31]。In IPF, fibroblast activation and excessive extracellular matrix deposition are hallmark features. The overexpression of *PDLIM7* may exacerbate these processes by influencing actin dynamics. Additionally, *PDLIM7* has been shown to interact with PDLIM2 to form heterodimers that suppress NF-κB-mediated inflammatory responses by ubiquitinating and degrading p65 [32]. The NF-κB signaling pathway plays a central role in fibrosis by promoting fibroblast proliferation and myofibroblast differentiation [33,34]. Furthermore, *PDLIM7* is involved in regulating cholesterol metabolism [35,36], which is critical for maintaining cell membrane integrity and intercellular communication, potentially contributing to fibrosis progression.

*CFAP45* is a protein essential for cilia formation and motility. Dysfunction of cilia has been implicated in respiratory diseases, including post-transplant lung infections, COPD, and pulmonary fibrosis [37–40]. Aberrant expression of cilia-related genes, such as *CFAP45*, in IPF may impair airway defense mechanisms and exacerbate fibrosis by affecting cilia-associated signaling pathways. *CFAP45* may also regulate cholesterol homeostasis in cell membranes by maintaining ciliary structure.

*HP* is an acute-phase protein primarily synthesized in the liver. It binds free hemoglobin to prevent oxidative damage and iron loss and is a major regulator of oxidative stress and inflammation [41]. Studies have shown that *HP* may influence macrophage activity and the NF-κB signaling pathway by binding to lipopolysaccharides [42]. Elevated *HP* expression in IPF patients may reflect an adaptive defense mechanism against persistent oxidative stress in the lungs. However, sustained *HP* upregulation could enhance the pro-fibrotic activity of macrophages and neutrophils, aggravating pathological changes.

The IPF prognostic model based on *PDLIM7*, *CFAP45*, and *HP* demonstrated excellent predictive performance in both training and validation cohorts. Kaplan-Meier survival analysis and ROC curve evaluations showed that patients in the high-risk group had significantly worse outcomes than low-risk group patients, with AUC values exceeding 0.70. This suggests the model’s robust discriminatory and predictive ability. Clinically, this model holds significant potential for application. First, it can aid in the early identification of IPF patients with poor prognoses, enabling clinicians to adopt more aggressive therapeutic strategies. Second, the risk model may serve as a basis for future nomogram development and clinical risk stratification in IPF.

The immune microenvironment plays a critical role in IPF progression. Immune infiltration analysis using the CIBERSORT algorithm revealed increased infiltration of neutrophils in the high-risk group, whereas monocytes were more prevalent in the low-risk group, suggesting that dysregulated cholesterol metabolism may promote IPF pathogenesis by modulating immune cell function. *PDLIM7*, *CFAP45*, and *HP* were significantly correlated with monocyte and neutrophil infiltration, further supporting the role of cholesterol metabolism in regulating immune cells. Cholesterol and its metabolites have been shown to influence macrophage polarization, neutrophil recruitment, and inflammatory responses [43–45].

Our single-cell analysis showed that epithelial cells and macrophages display altered abundance and distinct expression programs in IPF, suggesting functional involvement in fibrogenesis. Injured epithelial cells (especially AT2 cells) have impaired repair capacity and can release profibrotic signals (e.g., TGF-β/PDGF/WNT-related pathways), thereby activating fibroblasts and promoting collagen deposition. Meanwhile, macrophage subpopulations are reprogrammed toward a profibrotic phenotype, producing mediators such as TGF-β and CTGF and influencing ECM remodeling (e.g., via MMP/TIMP balance), which supports myofibroblast activation and scar formation.These cell-type changes help explain the bulk transcriptomic DEGs: bulk signals reflect both shifts in cell proportions (“composition effects”) and changes in cell states (“state effects”). Therefore, the expansion of injury-associated epithelial programs and profibrotic macrophage states is expected to drive the observed gene-expression differences and is consistent with progressive fibrotic scarring in IPF.

Constructing a ceRNA network provided insights into the potential regulatory mechanisms of *PDLIM7*, *CFAP45*, and *HP* in IPF. Several miRNAs and lncRNAs were predicted to bind these genes’ mRNAs competitively, influencing their expression in IPF. Cholesterol metabolism-related genes were identified as targets of multiple miRNAs, suggesting that regulating miRNA expression could indirectly modulate their activity and affect disease progression [46,47]. LncRNAs, as competitive endogenous RNAs, also play critical roles in cholesterol metabolism [48] and IPF progression [49]. compound prediction analyses identified several small molecules that may target cholesterol metabolism genes or related pathways, offering potential therapeutic options for IPF. Future in vitro and in vivo experiments are needed to validate the efficacy of these compounds and identify promising candidates for clinical translation.

However, several limitations should be noted. First, the GEO cohorts used for model development and validation (e.g., GSE150910 and GSE93606) are mainly derived from European and North American populations; thus, the generalizability of our findings to other populations requires further validation in larger, prospective, multi-ethnic cohorts. Second, bleomycin-induced lung injury model does not fully recapitulate the pathological changes observed in true idiopathic pulmonary fibrosis (IPF) lungs and our animal validation was limited to mRNA expression without protein-level confirmation (e.g., IHC/Western blot), which restricts mechanistic interpretation. Third, the predicted compounds are preliminary in silico candidates, and their regulatory directionality and mechanisms in IPF remain to be experimentally validated. Future studies will prioritize protein-level validation and functional experiments, and test candidate compounds in bleomycin-induced fibrosis models.

## 5. Conclusion

This study developed a prognostic model based on genes from a cholesterol metabolism–associated module, suggesting that cholesterol metabolism-related pathways may indirectly influence IPF progression through mechanisms involving cellular structure and immune regulation. *PDLIM7*, *CFAP45*, and *HP* were identified as key biomarkers that influence cellular migration, ciliary function, and oxidative stress, driving the pathological progression of IPF. Additionally, cholesterol metabolism’s regulation of the immune microenvironment, particularly macrophage polarization and neutrophil activity, provides novel therapeutic targets for metabolic intervention in IPF. Future studies should explore the complex interplay between cholesterol metabolism, immunity, oxidative stress, and fibrosis to develop more precise therapeutic approaches. While the model demonstrated strong generalizability in independent datasets (e.g., GSE28221), further validation with larger clinical cohorts is necessary to ensure its broad applicability. Integrating high-quality single-cell data and experimental validation will enhance our understanding of IPF’s key cellular interactions and molecular mechanisms.

## Supporting information

S1 Fig Primer sequences for RT–qPCR of CFAP45, HP, and PDLIM7, with GAPDH as the internal control. (JPG)

S2 Fig Validation of 13 Survival-Related Genes. *RAB36*, *ROPN1L*, *PDLIM7*, *F5*, *CFAP45*, *IL1R1*, *LRRC4*, *TSPAN2*, *NSUN7*, *S100P*, *SLPI*, *HP*, and *TCN1PH* (x-axis: survival time, y-axis: residuals generated by each covariate; solid lines are smoothed spline fits, dashed lines indicate ±2 standard deviations).
(JPG)

S3 Fig The differential immune cells between HRG and LRG were explored by the ssGSEA method. A. Heat maps of infiltration of 28 types of immune cells in HRG and LRG. B. The box plots of immune cells with significant differences in HRG and LRG were observed. ns indicates *P* > 0.05, * indicates *P* < 0.05, ** indicates *P* < 0.01, *** indicates *P* < 0.001, **** indicates *P* < 0.0001.
(TIF)

S4 Fig Quality Control and Analysis of Single-Cell Gene Expression. A. Gene expression distribution in cells before (upper) and after (lower) quality control (nFeature_RNA: number of genes detected per cell; nCount_RNA: count of gene expression per cell; percent.mt: proportion of mitochondrial genes). B. Selection of highly variable genes. C-D. PCA analysis and assessment of the top 30 PCs.
(JPG)

S5 Fig Heatmap of Communication Networks between Different Cell Types. (JPG)

## Abbreviations

IPF: Idiopathic pulmonary fibrosis
CMRGs: Cholesterol metabolism-related genes
scRNA-seq: single-cell RNA sequencing
WGCNA: Weighted Gene Co-expression Network Analysis
PPI: protein-protein interaction
DEGs: Differentially expressed genes
(K-M) curves: Kaplan-Meier curves
GO: Gene Ontology
KEGG: Kyoto Encyclopedia of Genes and Genomes
GSEA: Gene set enrichment analysis
miRNAs: microRNAs
lncRNAs: long non-coding RNAs
Ctrl: control
BLM: bleomycin
RT qPCR: Reverse transcription‑quantitative polymerase chain reaction
H&E: Hematoxylin and eosin
PDLIM7: PDZ and LIM Domain 7
CFAP45: Cilia and Flagella Associated Protein 45
HP: Haptoglobin
CC: cellular component
