# Supplementary figures and images for: Prognostic risk model based on cholesterol metabolism–Associated gene module for idiopathic pulmonary fibrosis
Source: PLoS One. 2026 Apr 9;21(4):e0345310. doi: 10.1371/journal.pone.0345310 (PMC13065012; doi:10.1371/journal.pone.0345310)

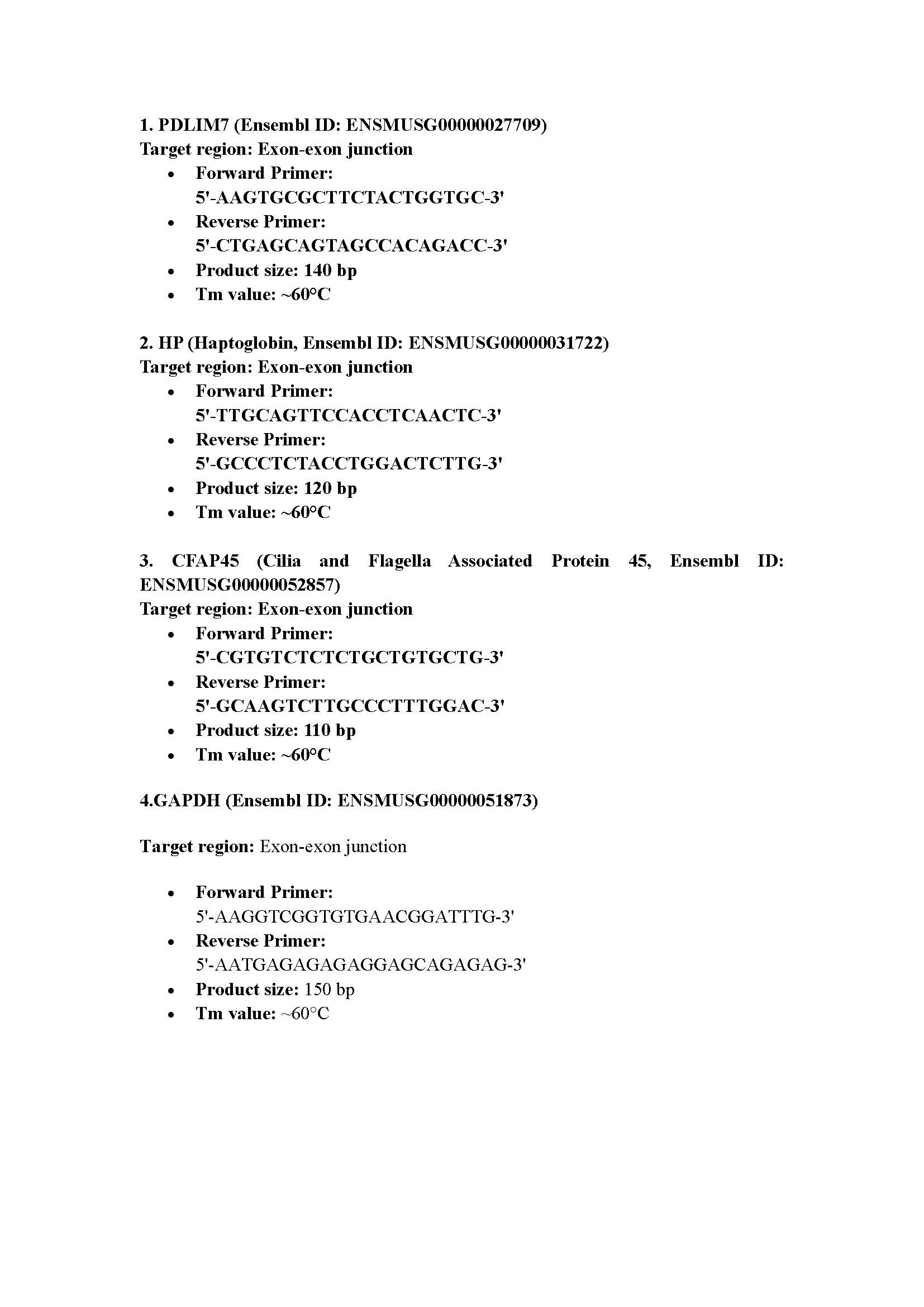

Supplement: S1 Fig — (JPG) [file pone.0345310.s001.jpg]

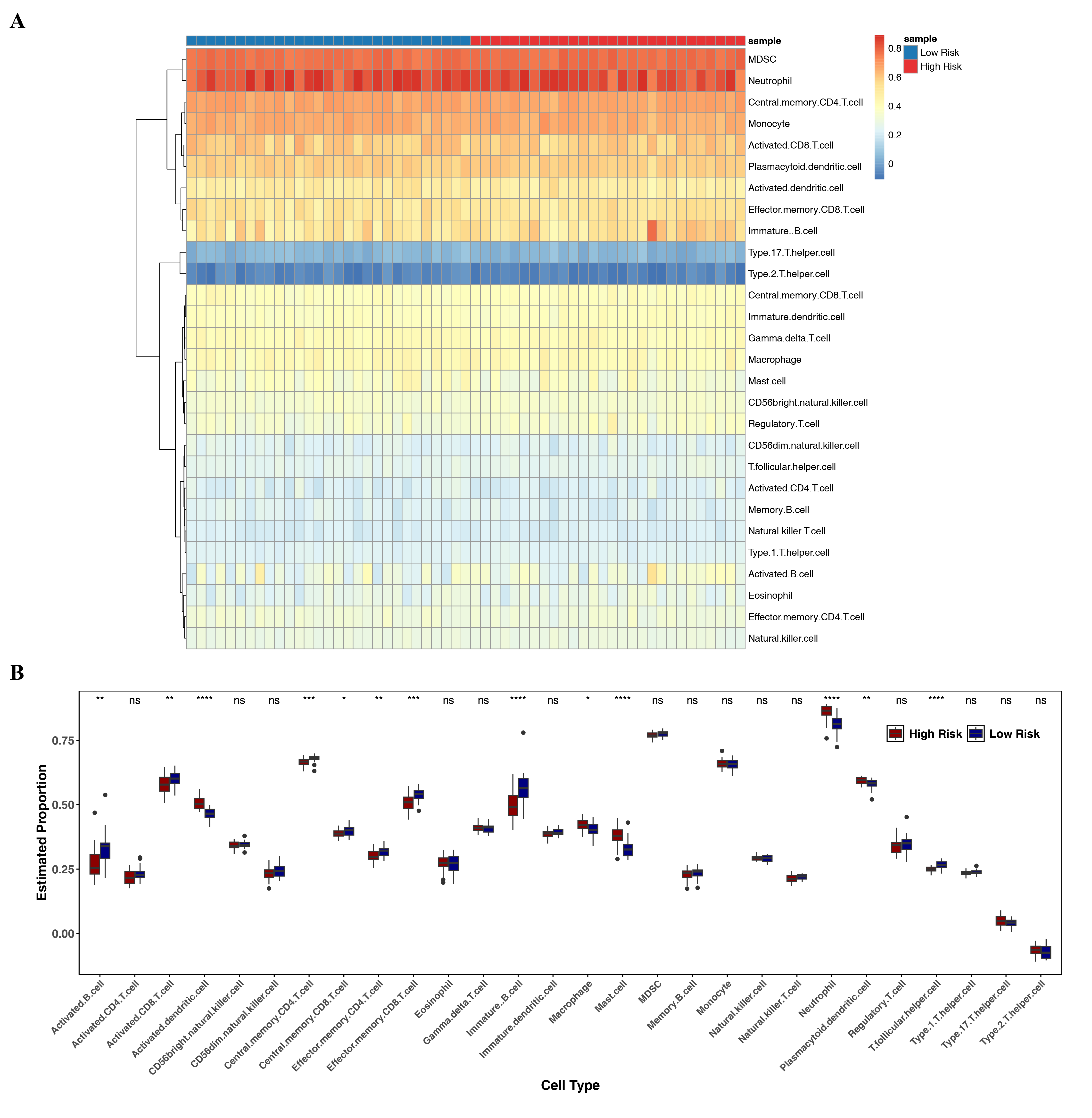

Supplement: S3 Fig — A. Heat maps of infiltration of 28 types of immune cells in HRG and LRG. B. The box plots of immune cells with significant differences in HRG and LRG were observed. ns indicates P > 0.05, * indicates P < 0.05, ** indicates P < 0.01, *** indicates P < 0.001, **** indicates P < 0.0001. (TIF) [file pone.0345310.s003.tif]
